# Inhibition of cardiac CaMKII to cure heart failure: step by step towards translation?

**DOI:** 10.1007/s00395-016-0582-1

**Published:** 2016-09-28

**Authors:** Friederike Cuello, Kristina Lorenz

**Affiliations:** 1 Department of Experimental Pharmacology and Toxicology, Cardiovascular Research Center, University Medical Center Hamburg-Eppendorf, Martinistrasse 52, 20246 Hamburg, Germany; 2 DZHK (German Center for Cardiovascular Research), Partner Site Hamburg/Kiel/Lübeck, University Medical Center Hamburg-Eppendorf, Martinistrasse 52, 20246 Hamburg, Germany; 3 Comprehensive Heart Failure Center, Versbacher Strasse. 9, 97078 Würzburg, Germany; 4 West German Heart and Vascular Center Essen, University Hospital Essen, Hufelandstrasse 55, 45147 Essen, Germany; 5 Leibniz-Institut für Analytische Wissenschaften-ISAS-e.V., Bunsen-Kirchhoff-Strasse 11, 44139 Dortmund, Germany

During the past decade, calcium/calmodulin-dependent protein kinase II (CaMKII) has emerged as a central culprit in the development of cardiac arrhythmia and heart failure. This has been supported by a plethora of studies using transgenic mouse models and pharmacological CaMKII inhibitors and peptides. However, the final proof that CaMKII inhibition improves dysfunction of a failing heart is still pending. In this issue, Kreusser et al. [19] demonstrated that knockdown of the two key cardiac CaMKII isoforms after the onset of hemodynamic stress succeeds to reverse maladaptive cardiac remodeling processes. Their study is an important step in translating CaMKII inhibition from bench to bedside for heart failure therapy.

CaMKII is a multifunctional protein kinase that plays a pivotal role in cardiac (patho) physiology [27, 40]. It represents a nodal point in the regulation of intracellular Ca^2+^ handling, ion channels and gene transcription. As the description “multifunctional” and “nodal point” implies, this kinase is intertwined into a complex cellular signaling network and thus tricky to manipulate therapeutically: CaMKII activity is regulated by posttranslational modifications that allow maintenance of kinase activity independently of Ca^2+^/calmodulin-binding. Amongst those autophosphorylation [16, 26, 45], oxidation [2, 7, 13, 32, 45], *O*-linked *N*-acetylglucosamination [9] and *S*-nitrosylation [8, 12] have been described to date. CaMKII has various cellular targets in Ca^2+^ homeostasis some of which are the ryanodine receptor [26, 34, 39, 41], phospholamban [5, 18] and cardiac myosin-binding protein C [37]. Furthermore, CaMKII impacts on L-type Ca^2+^ channel (LTCC) currents and LTCC expression [40], on expression of the Na^+^/Ca^2+^ exchanger [10, 23] and the sarcoplasmic reticulum Ca^2+^ ATPase 2a (SERCA2a) [5, 23, 42] as well as on gene transcription via the regulation of calcineurin and class II histone deacetylase isoforms [3, 24]. CaMKII is ubiquitously expressed, with α and β as predominant isoforms in the brain, where they are important for neuronal function and cognitive memory. CaMKIIγ and δ are the key isoforms expressed in the heart. Particular attention has been paid to two CaMKIIδ splice variants in the heart, CaMKIIδ_B_ and CaMKIIδ_C_. CaMKIIδ_B_ has an 11 amino acid nuclear localization sequence that is absent in CaMKIIδ_C_. Studies performed in splice-variant-specific knockout mouse models have attributed a protective functional role to CaMKIIδ_B_. Thus, cellular localization seems to participate in CaMKII isoform-specific pathophysiological roles [4, 5, 28, 44].

Despite of the physiological importance of CaMKII, for e.g,. excitation–contraction coupling, isoproterenol-induced heart rate adaptation, cognitive memory and neural plasticity functions, inhibition of CaMKII as a therapeutic strategy in different forms of cardiac disease increasingly solidifies. Cardiac expression and activity of CaMKII have been shown to be increased in cardiac disease and more importantly also augments the incidence of cardiac disease, particularly arrhythmia, atrial fibrillation and progressive cardiac remodeling [6, 15, 17, 21, 27, 29, 42, 43]. Proof-of-concept studies in mice and isolated human cardiac myocytes have successfully demonstrated the benefit of CaMKIIδ and CaMKIIγ inhibition in several pathological cardiac conditions. However, translation of these convincing preclinical “prevention” studies into therapeutic strategies or even a preclinical “therapy/rescue” study seems rather challenging.

Difficult hurdles that have to be envisaged to selectively target disease-specific kinase functions [22, 38] to ultimately achieve clinical translatability are the design of (1) appropriate cardiac-specific gene therapy approaches for the expression of inhibitory peptides, proteins or knockdown-vectors or (2) isoform-specific, orally administrable, non-CNS penetrating small compound inhibitors. The peptides and compounds that have been developed thus far have been shown to exert off-target effects, which include the inhibition of potassium channels (KN-93; [14]), blockade of anchoring proteins and substrates (CaMKIIN and CaMKIINtides; [25, 30]) and in case of ATP-competitive compounds the inhibition of other kinases (SMP-114; [30]), or they are orally not bioavailable [30].

Pharmacological CaMKII inhibitors as well as the transgenic mouse models have been extremely valuable for dissecting the functional roles of CaMKII in cells and in vivo, but they remain experimental tools (reviewed in [29, 30, 42]). And, the question, whether CaMKII inhibition—in an ideal “off-target free” setting—has the potential to ameliorate cardiac remodeling and cardiac function after the onset of heart failure, is still unanswered. With the tools available, this question was not yet appropriately addressable. Kreusser et al. [19] have now developed genetic mouse models with inducible CaMKIIδ and γ knockdown to address exactly this issue: “Is CaMKII inhibition able and sufficient to rescue a failing heart?” And the answer from this mouse study is “Yes”! This is the first time that CaMKII inhibition has been tested in a “therapy/rescue” situation.

In their study, the authors use mouse models that are based on a cardiac-specific conditional knockdown of CaMKIIδ and γ by tamoxifen or by Cre-recombinase overexpression via adeno-associated viral vectors. In both approaches, the development of interstitial fibrosis and contractile defects in response to chronic left ventricular pressure overload (induced by transverse aortic constriction) was decelerated and even slightly reversed. This study shows convincingly that inhibition of cardiac CaMKII expression is a promising goal for the improvement of chronic heart failure therapy. Thus, the effort has to be taken and to be enforced to realistically name CaMKII a clinically relevant target.

However, it will still be a long and arduous way to implement clinical CaMKII inhibition for heart failure therapy. Due to the high homology between existing CaMKII isoforms and their physiological roles, CaMKII targeting strategies have to take cardiac and isoform specificity into account. In this context, the establishment of a gene therapy approach seems “easier” than the development of pharmacological inhibitors. Also, gene therapy studies in large animals or even patients have already been tested for S100A1, SERCA2a and adenylyl cyclase 6 [1, 11, 20, 31, 33, 35, 36]. SERCA2a gene therapy has already reached clinical phase IIb studies, but then failed due to insufficient delivery of viral particles to the heart. Nevertheless, these studies delivered proof of the general concept, and have at the same time revealed the difficulties that still need to be overcome. For the development of small pharmacological compounds, even more challenges have to be faced: As mentioned before, the CaMKII family comprises highly homologous isoforms and splice variants, which makes selective pharmacological targeting of a specific isoform or splice variant rather impossible. At this point, the manipulation of certain downstream targets of CaMKII comes into play. Thus far, however, it is not clear, which of the targets has the major impact on cardiac disease progression or if there are even targets of different importance in different cardiac diseases. It will be a major effort to dissect the impact of the CaMKII-mediated molecular effects in different disease scenarios to really be able to predict the therapeutic benefit of target specific CaMKII inhibition. To promote the design of an appropriate pharmacological compound, we will certainly have to disentangle physiological from the pathological CaMKII functions. With their study Kreusser et al. [19] have clearly demonstrated that CaMKII inhibition is the right avenue to tread for significant benefit in heart failure therapy in the future.
